# Bibliometric analysis of global research profile on ketogenic diet therapies in neurological diseases: Beneficial diet therapies deserve more attention

**DOI:** 10.3389/fendo.2022.1066785

**Published:** 2023-01-04

**Authors:** Yinuo Wang, Junyao Zhang, Yingying Zhang, Junyan Yao

**Affiliations:** Department of Anesthesiology, Shanghai General Hospital, Shanghai Jiao Tong University School of Medicine, Shanghai, China

**Keywords:** Ketogenic Diet, Neurological Diseases, bibliometric analysis, hotspots, Citespace, VOSviewer, Oxidative Stress, Neuroprotection

## Abstract

**Background:**

The protective effects of Ketogenic Diet Therapies (KDTs) on neurological diseases have been extensively studied over the past two decades. The purpose of this study was to quantitatively and qualitatively analyze the publication of KDTs in the neurological field from 2000 to 2021.

**Methods:**

A literature search was performed on June 7th, 2022, using the search terms: ((“ketone” OR “ketogenic” OR “*hydroxybuty*”) AND (“neuro*”)) in the WoSCC database. Collected data were further analyzed using VOSviewer, CiteSpace and other online bibliometric websites. The annual publication volume and citation trends were summarized. The collaborations among highly cited countries, institutions, authors and journals were visualized. The co-citation analysis of highly cited references and journals were also visualized. Moreover, the research focuses and fronts were revealed by co-occurrence analysis and burst keywords detection.

**Results:**

A total of 2808 publications with 88,119 citations were identified. From 2000-2021, the number of publications and citations presented rising trends. *The United States* was the country with an overwhelming number of publications and cited times. *Johns Hopkins University* was the most contributory institution. *Kossoff Eric H* was the author with the largest number of publications. And *Epilepsia* was both the largest publisher and the most frequently cited journal. The keywords of intense interest involved “Modified Atkins Diet”, “Temporal Lobe Epilepsy”, “Alzheimer’s Disease”, “Parkinson’s Disease”, “Cerebral Blood Flow”, “Neuroinflammation”, “Oxidative Stress”, “Metabolism” and “Mitochondria”.

**Conclusion:**

We presented the global trend of KDTs in neurological diseases and provided important information for relevant researchers in a bibliometric way. This bibliometric study revealed that treating epilepsy, neuroprotection and functional effects of KDTs on mitochondria and oxidative stress have been the spotlight from 2000 to 2021. These have emerged as the basis for transformation from basic research to clinical application of KDTs.

## 1 Introduction

Ketogenic Diet Therapies (KDTs) are high-fat, low-carbohydrate and moderate protein diets that induce the production of ketone bodies through fat metabolism. Usually, KDTs can simulate a fasting state without depriving the calories body need for growth and development (1, 2). KDTs influence the body beyond drugs and fundamentally change how the body takes in energy (3, 4). Therefore, KDTs may exert an irreplaceable role in improving the disease through affecting body metabolism, which make them important for treatment.

The classical ketogenic diet (KD) has initially been proved effectively in treatment of children with intractable epilepsy in 1920s. Nowadays, multiple modified KDTs are gradually replacing the classical KD i.e. the medium chain triglyceride (MCT) ketogenic diet, the modified atkins diet (MAD), the low glycemic index treatment (LGIT) (5).

KDTs therapeutic benefits have also been proven in many other neurological diseases (6–10). Their neuroprotective mechanism and clinical application have been constantly explored since 2000. KDTs may manipulate brain energy metabolism in chronic neurological diseases to provide neurological benefits (11). While in acute illnesses, KDTs prefer to inhibit neurological inflammation (6). Although numerous studies have been developed, there is a lack of comprehensive systematic analysis and progressing report of KDTs in neurological diseases.

Bibliometric analysis has become a powerful tool for quantitative analysis of academic literature within a particular field including medical sciences (12). Therefore, to better understand the current profile of KDTs, a bibliometric analysis to evaluate the research progress and analyze the status is necessary for more comprehensive understanding of KDTs in neurological diseases. Herein, we recapitulated the developmental trends of KDTs based researches in neurological disease by visualizing the cooperation network in different dimensions, summarizing the main research clusters, hotspots and keywords and analyzing the co-occurrence to provide hints for the researchers to further explore unsolved problems of this field in the future.

## 2 Methods

### 2.1 Data source and search strategies

**Figure 1:**
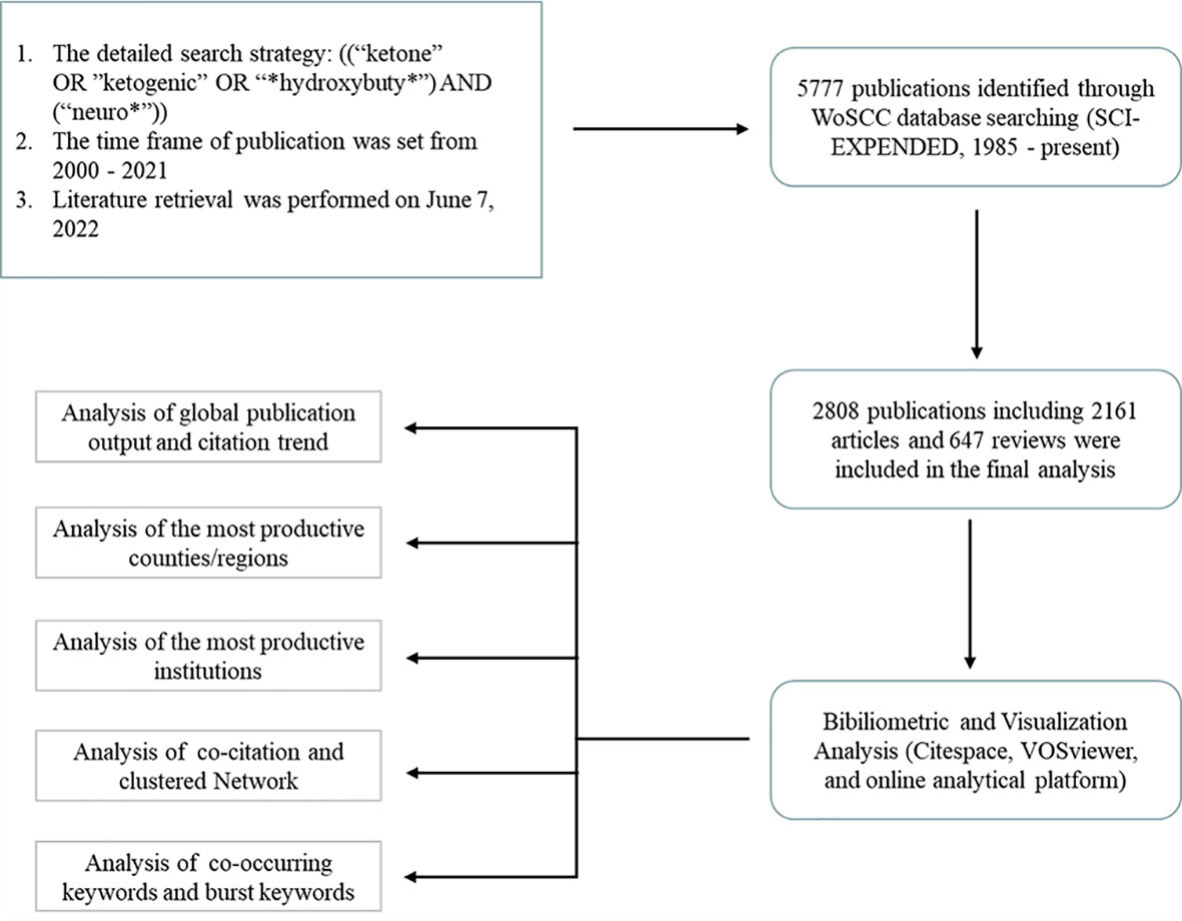
Flowchart of the literature searching and publication screening (* is a wildcard character).

Data of bibliometric analysis were conducted using the Web of Science Core Collection (WoSCC) database (Clarivate Analytics, Philadelphia, PA, USA). Literature retrieval was performed on June 7th, 2022 by two authors (Yinuo Wang and Junyao Zhang) independently to obtain the initial data to eliminate the bias led by daily updates of the database. The detailed search strategies were as follows: ((“ketone” OR “ketogenic” OR “*hydroxybuty*”) AND (“neuro*”)) with a limited time frame set from 2000 to 2021. Language type was restricted to English and the publication type was limited to original articles and reviews. **Figure 1** presented the literature search and selection process.

This study was based on the WoSCC database and did not involve direct interaction with human participants. Hence, ethical approval was not required.

### 2.2 Data extraction and collection

Following the search strategies described above, all retrieved literature was downloaded and exported in text format for further analysis. Then, the records including titles, authors, abstracts and cited references were exported to detection software to remove duplicates. Subsequently, WoSCC database, Microsoft Excel 2019 (Microsoft Corporation), VOSviewer (version 1.6.18), CiteSpace (version 6.1.R2), online analytical platform (https://charticulator.com) and R package (version 4.2.1) were used to conduct the bibliometric analysis. The annual publications and citations were collected in the WoSCC analysis tool. Data were extracted from the WoSCC database to identify collaborations among highly influential countries, institutions, authors, references and keywords using VOSviewer. CiteSpace was used to perform co-reference analysis, journal dual-map overlay analysis and burst keywords visualization. The heatmap of keywords was generated by R package.

## 3 Results

### 3.1 Analysis of KDTs global publication output and citation trend

**Figure 2:**
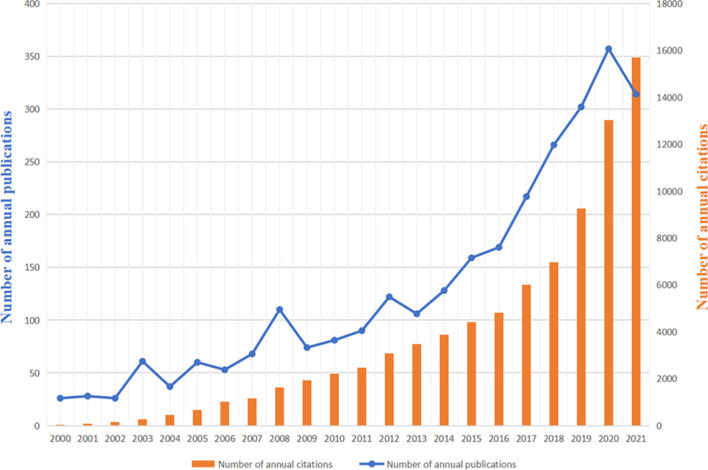
Global trends of annual publications and citations from 2000 to 2021.

2808 publications matching the retrieval criteria were included for further bibliometric analysis (**Figure 1**). The cumulative citations for all publications from 2000 to 2021 were 89,899 times and the average number of citations per item was 31.7 with an H-index of 122. Trends of annual publication volume and citation frequency were visualized in **Figure 2**. Since 2000, annual publications have been constantly increasing every year. The number of annual publications showed an overall upward trend from 2000 to 2020. There were three small peaks in 2003, 2008 and 2012. Furthermore, in 2020, the number of publications reached an all-time peak. In 2021, it declined to a certain extent compared to 2020 due to the late publication time. As for annual citations, from 2000 to 2016, there was a relatively stable and slight increase. The number of annual citations experienced a blowout after 2016.

### 3.2 Analysis of the most productive countries/regions of KDTs research

A total of 90 countries have published relevant researches and countries publishing more than five publications were included. A total of 53 countries met the criteria, of which the publications, citations and total link strength of the top 10 countries were shown in **Table 1**.

**Table 1:** List of top 10 countries of Ketogenic Diet studies.

Country	Documents	Citations	Total Link Strength
***The United States***	1,151	48,125	606
***China***	264	5,151	108
***Germany***	208	6,188	320
***England***	205	8,503	377
***Italy***	204	6,421	248
***Canada***	181	6,024	224
***Japan***	151	4,545	96
***France***	134	4,726	214
***Australia***	107	3,407	188
***Spain***	97	2,175	112

**Figure 3:**
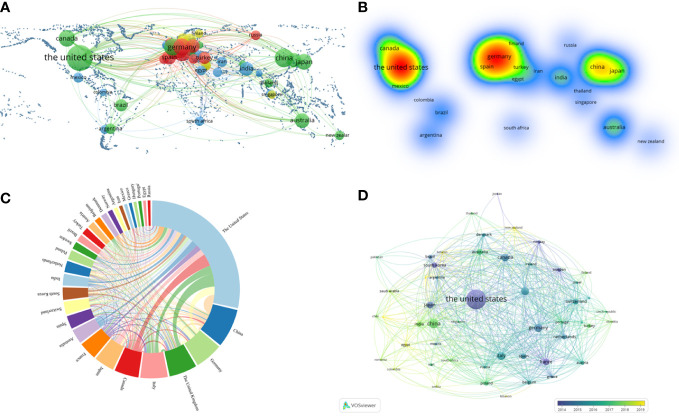
VOSviewer and online analytical platform analyses of collaborations among different countries. **(A)** A world map displaying the geographical distribution of researches generated by an online analytical platform. **(B)** A world heat map demonstrating the geographical intensity of researches generated by VOSviewer. **(C)** The visualization map of publication and collaboration among countries generated by an online analytical platform. The thickness of each line reflected the tightness of cooperation, and the thicker the line indicated the more vital the collaboration. **(D)** Country co-authorship overlay visualization map classified by study time generated by VOSviewer. The lighter the color represented the more recent the study.

In the geographic visualization, all publications among 53 countries with the same color represented the same cluster (**Figures 3A, B**). The United States, Canada, Australia, New Zealand, Japan and China belonged to the green cluster. Germany, Turkey, Spain and Russia belonged to the red cluster. Similarly, India, Iran, South Africa and Mexico belonged to the blue cluster. In comparison, Finland and Singapore belonged to the yellow cluster. And The United States and Germany were cores of the heatmap (**Figure 3A, B**). The United States had the most significant number of publications and extensive cooperation with almost every country. China placed second, Germany and the United Kingdom followed closely regarding publication (**Figure 3C**).

A country co-authorship overlay visualization map was also generated (**Figure 3D**). In this map, the lighter the color, the more recent the study. So, the purple nodes represented that most of the publications from the United States were around 2014. Whereas, the nodes in Germany, Canada and other countries were mainly blue, which meant that their publications were mainly located in 2015-2016. And the node in China was green, which indicated that its publications were mainly located in 2017-2018.

### 3.3 The most prolific institutions of KDTs studies

**Figure 4:**
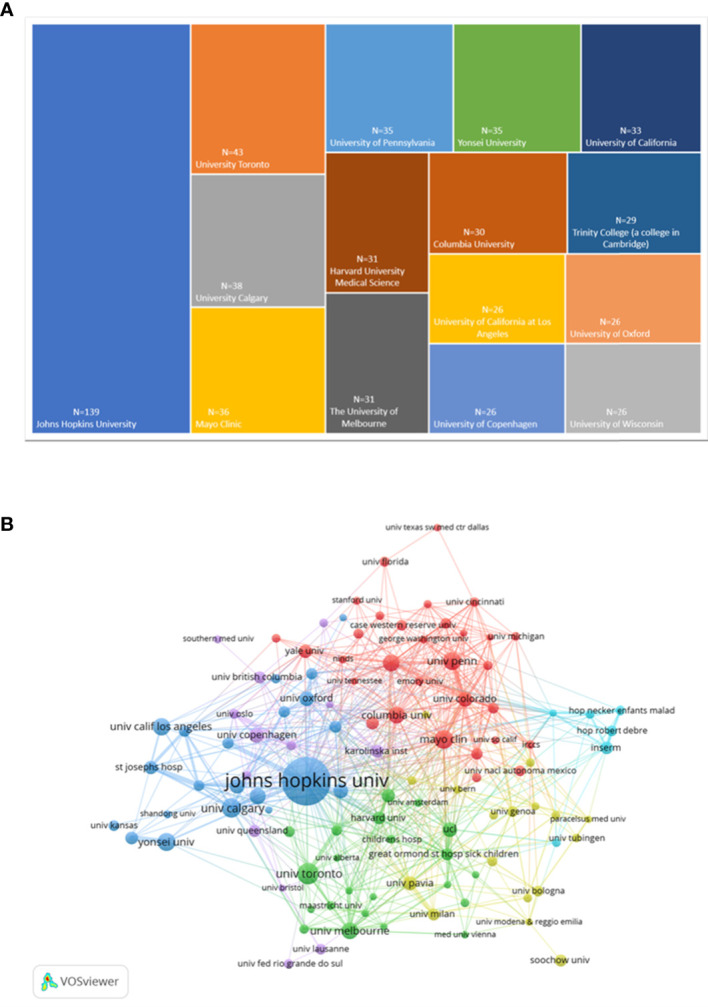
Analysis chart of the most prolific institutions. **(A)**The top 15 institutions with the greatest number of publications presented by Excel.**(B)**Institution co-authorship overlay visualization map generated by VOSviewer.

The institutions with the top 15 highly publications were presented (**Figure 4A**). Among them, *Johns Hopkins University* held the most significant number of publications with 139, followed by *University Toronto* and *University Calgary* with 43 and 38.

112 institutions were included, with the selection criteria of a minimum publication volume of 10, and were grouped into five clusters (**Figure 4B**). *Johns Hopkins University* was the institution with the highest citations, followed by *Niagara University* and *Barrow Institute of Neurology*. In terms of total link strength, the most essential institution was *Johns Hopkins University*, followed by *Mayo Clinic* and *Harvard Medical School*. The top 10 institutions with their publications, citations and total link strength were shown in **Table 2**. In addition, the top 10 authors and journals with their publications, citations and total link strength were shown in **Table 3**, **4**.

**Table 2:** List of top 10 organizations of Ketogenic Diet studies.

Organization	Documents	Citations	Total Link Strength
***Johns Hopkins Univ***	139	7737	81
***Univ Toronto***	43	1198	28
***Univ Calgary***	38	1487	37
***Mayo Clinic***	36	1678	63
***Univ Pennsylvania***	35	1518	45
***Yonsei Univ***	35	1309	19
***Univ Calif Los Angeles***	33	1939	10
***Univ Melbourne***	31	1113	38
***Harvard Med Sch***	31	620	53
***Columbia Univ***	30	1755	37

**Table 3:** List of top 10 authors of Ketogenic Diet studies.

Author	Documents	Citations	Total Link Strength
***Kossoff Eric H***	57	2454	2004
***Rho Jong M***	46	2900	2030
***Cross J Helen***	29	1317	883
***Kim Heung Dong***	26	830	577
***Masino Susan A***	25	1019	993
***Cervenka Mackenzie C***	22	559	691
***Hartman Adam L***	19	1547	1164
***Kang Hoonchul***	19	594	395
***Turner Zahava***	19	618	639
***Cunnane Stephen C***	18	627	594

**Table 4:** List of top 10 journals of Ketogenic Diet studies.

Journals	Documents	Citations	Total Link Strength
***Epilepsia***	151	7875	2695
***Epilepsy Res***	108	2694	1668
***J Child Neurology***	68	1689	878
***Nutrients***	53	638	782
***Brain & Development***	44	749	309
***Plos One***	41	1714	463
***J Neurochemistry***	40	2622	708
***European J Paediatric Neurology***	33	936	343
***Developmental Medicine and Child Neurology***	32	1159	429
***Epilepsy & Behavior***	28	386	335

### 3.4 Analysis of co-citation and clustered network

**Figure 5:**
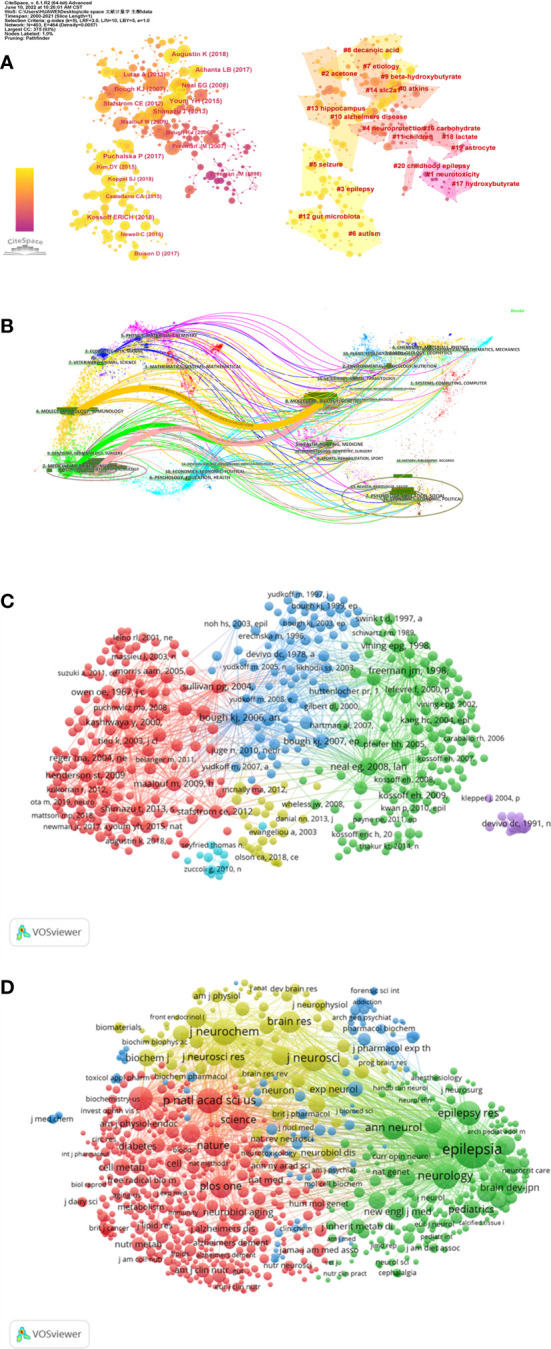
Co-citation map of references generated by CiteSpace and VOSviewer. **(A)** A Journal co-citation network map and the clustered network map of co-cited references generated by CiteSpace. **(B)** The dual-map overlay generated by using CiteSpace. **(C)** Network visualization map of cited references analysis generated by the VOSviewer. **(D)** Network visualization map of cited sources analysis generated by the VOSviewer.

The co-citation and clustered network maps were generated by CiteSpace from 93,213 references in a hierarchical order (**Figure 5A**). Visualization of co-cited references showed a total of 875 nodes and 4,523 links. In this network, each node represented a cited publication, and the size of each node was proportional to the total co-citation frequency of the associated publication. The co-cited references were clustered into cluster labels and 20 crucial labels were presented including Atkins, neurotoxicity, acetone, epilepsy, neuroprotection, seizure, autism, etiology, decanoic acid, beta-hydroxybutyrate, Alzheimer’s disease, children, gut microbiota, hippocampus, slc2a1, carbohydrate, hydroxybutyrate, lactate, astrocyte and childhood epilepsy. The lighter the color represents the more recent the study. As reported, seizure, epilepsy, gut microbiota, autism, beta-hydroxybutyrate and decanoic acid were widespread these years.

The dual-map overlay of co-citation journals was shown through CiteSpace, the left part was the citing journals and the right part was the cited journals (**Figure 5B**). The broader lines that began from the citing journals and ended at the cited journals represented the main citing pathways calculated from the so-called z-score of the citation links.

The cited references and journals about this topic were visualized through VOSviewer. 605 references were selected from 106,829 references and grouped into 6 clusters. The selection standard was that a cited reference’s minimum number of citations was 20 (**Figures 5C, D**). Among them, the 2008 *Lancet Neurol* “doi10.1016/s1474-4422 (08) 70092-9” was the most influential publication with 284 citations and 5,304 total link strength, followed by 1998 *Pediatrics’s* “doi10.1542/peds.102.6.1358” and 2006 *Ann Neurol* “doi10.1002/ana.20899”. As for cited journals, the selection standard was 30 minimum number of journal citations. 753 journals were selected from 10,332 journals and grouped into 4 clusters. *Epilepsia* had the largest citations, followed by *Neurology*, *J Neurochem*, *J Neuroscience*, and *P Natl Acad Sci USA journals*.

### 3.5 Analysis of co-occurring keywords and burst keywords

**Figure 6:**
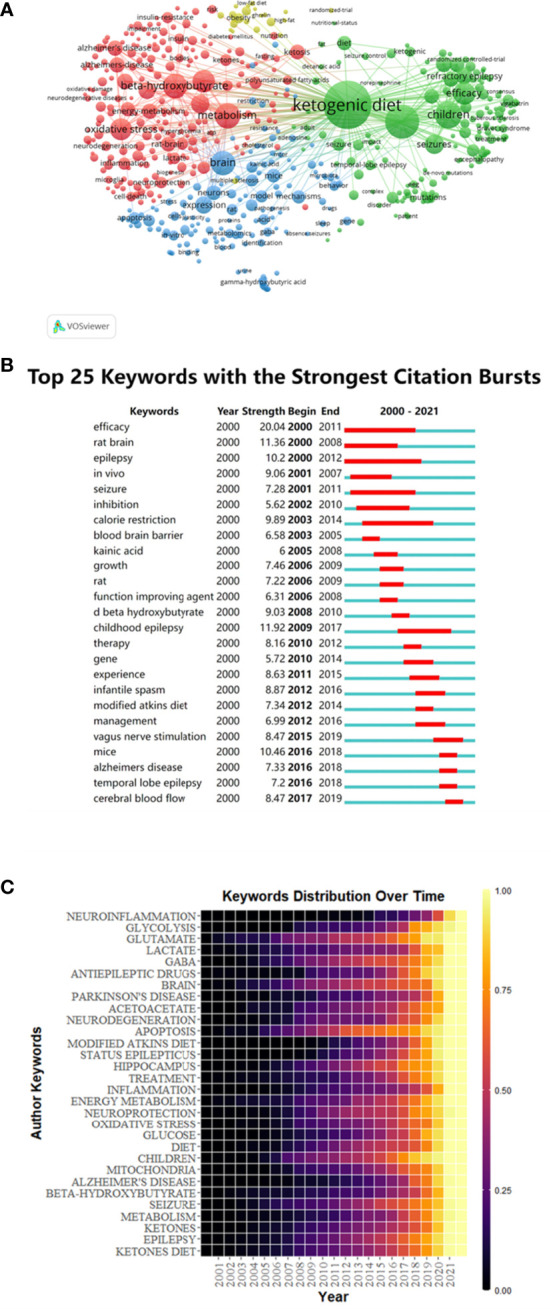
Keywords co-occurrence analysis of global research about this topic. **(A)** Mapping of keywords in the research generated by VOSviewer. **(B)** Keywords with the most robust citation bursts in original publications between 2000 and 2021 generated by CiteSpace. **(C)** Heat map of hotspots evolution of keywords generated by R package.

499 keywords were selected from 109,57 keywords with a standard of 10 minimum number of occurrences of a keyword. The top 6 keywords with the most frequent occurrences were as follows: ketogenic diet (1228), epilepsy (608), beta-hydroxybutyrate (331), children (385), metabolism (341), brain (316) and oxidative stress (256) (**Figure 6A**). The size of the points represented the keyword occurrence frequency and the keywords were divided into four clusters.

Burst keywords were important indicators of research frontiers, which indicated possible hotspots of future studies. We generated burst keywords between 2000 and 2021 through CiteSpace to identify the emerging concepts that have drawn attention of peer investigators (**Figure 6B**). The timeline was depicted as a year-sliced blue line, and the time interval of a burst was marked as a red section on the blue timeline to indicate the beginning/ending year and the duration of a citation burst. From 2001 to 2020, burst strength was the highest for efficacy (20.04), followed by rat brain (11.36), epilepsy (10.2), *in vivo* (9.06), seizure (7.28) and inhibition (6.62). Since 2003, the focuses have been on calorie restriction (9.89), followed by blood brain barrier (6.58) and kainic acid (6). Since 2006, some burst keywords, such as growth (7.46), rat (7.22), function improving agent (6.31) and D-β-hydroxybutyrate (9.03) continued for only a short period. Childhood epilepsy (11.92) has lasted for a long duration of time since 2009 to 2017. In addition, vagus nerve stimulation (8.47), mice (10.46), Alzheimer’s disease (7.33), temporal lobe epilepsy (7.2) and cerebral blood flow (8.47) have gradually become the most popular burst keywords.

The time distribution of keywords was also presented as a heatmap by R package (**Figure 5C**). The lighter the color, the more frequently the keyword has occurred. Before 2005, fewer studies were related to the KD and the nervous system. After 2005, the studies related to glutamate, GABA, brain, apoptosis, diet, children, seizure, and epilepsy gradually increased and they have once become hotspots in this field. While the studies related to neuroinflammation, lactate, Parkinson’s disease, neurodegeneration, modified atkins diet, inflammation, oxidative stress, Alzheimer’s disease and beta-hydroxybutyrate were emerging in the more recent years, indicating that these branches might be new hotspots and deserved further discussion.

## 4 Discussion

In this study, we conducted a multidimensional and comprehensive bibliometric analysis of the English articles and reviews between 2000-2021. We tried to reveal the current status of KDTs intuitively and provide hints for future research on this topic.

### 4.1 Research Trends of KDTs in neurological diseases

The classical KD initially emerged in 1921 for children with epilepsy, while its application has gradually decreased since 1970 with PubMed listing only two to eight publications per year from 1970 to 2000 (3). However, it has suddenly received global attention since 2000 when NBC-TV’s Dateline aired a program on its treatment effects (3, 13). It revealed that the increase of the ketone body in brain alters the endocrine states of the neurological system. In normal conditions, glucose is the primary fuel utilized by tissues (14). While in states of prolonged fasting (> 3 days), the counter-regulatory actions of glucagon, epinephrine and cortisol stimulate the mobilization of free fatty acids from the stored triglycerides in adipose tissue by their movements on lipoprotein lipase (14). These free fatty acids release into circulation undergo β-oxidation in the liver to form acetyl coenzyme A, which are then able to enter the citric acid cycle for complete metabolism *via* the oxidative phosphorylation (15). Simultaneously, when insulin is low and intracellular ATP is sufficient, these acetyl groups can be converted to ketone bodies for export from the liver (15). In conclusion, after the coverage of the TV program, there have been a surge of academic researches into KDTs.

Nowadays, remarkable therapeutic outcomes of multiple KDTs have been achieved for a wide range of neurological diseases other than epilepsy (7–10, 16–20). KDTs mainly include classical KD, MCT diet, MAD, LGIT, low carbohydrate ketogenic diet (VLCKD), Deanna protocol (DP) and Ketone Esters (KEs) diet (5, 14, 21). The MCT diet is proposed with 60% of calories from octanoate and decanoate that are more ketogenic than long-chain triglycerides (21).While, the MAD consists of a nearly balanced diet (60% fat, 30% protein, and 10% carbohydrates by weight), without the restriction of recommended daily calories according to patient age (6). The LGIT is characterized by higher amounts of carbohydrates with low glycemic index (5). VLCKD recommends 20 to 50g per day or 10% of a 2000 kcal/day diet to achieve a ketogenic state (14). DP is a metabolic therapy that provides alternative, energetic fuels such as ubiquinol, MCTs, and gamma-aminobutyric acid (21). And KEs mainly include 1, 3-butanediol monoester of βOHB and glyceryltri-3-hydroxybutyrate, which are suitable for oral treatment (21).

Their applications in neurological diseases other than epilepsy are currently being tested through a variety of disease models. In 2003, *Tieu* et al. reported the protective effects of the classic KD in Parkinson’s disease (22); in 2006, *Zhao* et al. demonstrated that classic KD, MCT and DP diet are beneficial to the functional recovery of Amyotrophic Lateral Sclerosis (23); the protective effects of KD and MCT in Rett syndrome were also confirmed in 2009 by *Mantis* et al. (24); in 2013, *Beckett* et al. and *Streijger* et al. proved that classical KD can improve energy metabolism and functional recovery in Alzheimer’s disease and spinal cord injury, respectively (25, 26); classical KD can improve the early motor-behavioral outcome after stroke was also confirmed in 2019 by *Shaafi* et al (27).

Generally, the number of publications and trends in the literature each year reflected the research development and progress. According to our bibliometric analysis (**Figure 2**), the scientific output and researchers dedicated to this field have been increasing continuously, which reflected the growing interest in this field. Although the quantity of researches was relatively sizable, critical analysis of research hotspots was still lacking. In this study, we categorized the properties of relevant researches and focused on the interpretation of keyword co-occurrence and burst detection in order to give some hints to future research trends.

Our result suggested that the United States owned an overwhelming number of literature publications, followed by China and Germany (**Table 1**). Their citations per document were 41.81, 19.51 and 29.75 respectively. It was found that the number of neuroscience publications in each country were directly proportional to their total per capita health expenditure (28). Therefore, it is not surprising that they have become the world leaders in this research field. Furthermore, all the top 10 highly cited institutions belonged to the United States and more than half of the most frequently and highly cited authors were also from the United States (6/10). In conclusion, The United States owned the most significant number of famous universities and led the tide of KDTs research around the world. China ranked second in the number of publications published, but the citations per document were only 19.51, which signified the need to improve the quality of researches.

As shown in **Table 2**, the top ten institutions were all located in the United States. As reported, *Johns Hopkins University* was the institution with overwhelming cited times among all these institutions, reflecting its authority in this field. Furthermore, the location relationship among institutions was relatively concentrated (**Figure 4**), indicating their academic cooperation and communication among institutions were quite close. In previous studies, some scholars suggested that more inter-agency communication and authors’ cooperation were likely to enhance research productivity and the quality of studies (29).

In the field of authors, *Kossoff Eric H*, *Rho Jong M*, *Cross J. Helen*, *Kim Heung Dong*, *Masino Susan A*, *Cervenka Mackenzie C*, *Hartman Adam L*, *Kang Hoonchul*, *Turner Zahava*, *Cunnane Stephen C* and many other authors have all made impressive achievements, influencing the research trends and current understanding in this field (**Table 3**). Among them, Professor *Kossoff Eric H* was an authoritative expert in KDTs therapy from *Johns Hopkins University*, the United States. He has made pioneering explorations of KDTs in neurological diseases such as glioma and epilepsy, which have been cited by numerous articles (30–34).

Analysis of literature sources can help researchers to find the core journals in their field. Our data suggested that the top 10 highly co-cited journals all placed emphasis on basic research of neurological system (**Table 4**). Therefore, we believed that KDTs were converting from clinical trials to basic researches and might pave the way for future clinical transformation. It was worth noting that *Epilepsia*, though had a lower impact factor compared to these top journals, was both the largest publisher and the most frequently cited journal, suggesting its therapeutic effects in epilepsy were still most wildly studied in this field. Even though *Lancet Neurol* was not one of the top 10 journals, we noticed that it had the most influential article with 284 citations and 5,304 total link strength, reflecting the authority of this journal from another point of view (**Figure 5**).

Moreover, publications from the top co-cited references could be used as authoritative references. Co-cited references provided crucial information regarding intellectual connections among various scientific concepts. Most of the top ten cited articles were from the randomized controlled trial and reviews, focusing on the clinical application, protective mechanisms and metabolic effects of KDTs in neurological diseases.

As shown in **Figure 5B**, the cited literature was published earlier and the citing literature was published later. As the graph demonstrated, the cited publications came from a wider variety of journals, including molecular, biology, genetics, psychology, education, social, health, nursing, medicine, environmental, toxicology, nutrition et al., while the sources of the citing publications were more concentrated, mainly from molecular biology, immunology or medicine journals. To some extent, it reflected the evolution of research in the field of KDTs, which started with multiple disciplines containing both natural and social sciences and was then concentrated on certain areas mainly concerning health and molecular mechanism, indicating hotspots of KDTs researches of today and the future.

### 4.2 Research focus on KDTs in neurological diseases

The clustering and co-citation analysis indicated that the treatment of epilepsy, neuroprotection of KDTs and functional effects of KDTs on metabolism were high-frequency topics from the top 10 highly cited references, reflecting the focuses in this field (**Figure 5**). Therefore, in the following discussion, we particularly introduced these hot topics, which provided valuable insights into the current stage of the research.

#### 4.2.1 Treatment of epilepsy

*Wirrell E* et al., Lambrechts E et al. and *Lyons L* et al. found that KD is more effective than many new anticonvulsants in treating children with exceptional uncontrolled seizures and is more acceptable to children (35–37). Thus, KD tends to be considered as replacement treatment for epilepsy. This is the initial application of KDTs in the treatment of neurological diseases.

#### 4.2.2 Neuroprotection of KDTs

After that, *Kashiwaya Y* revealed that additional D-β-hydroxybutyrate supplement protects cultured mesencephalic neurons from membrane palmitoylated protein 1 toxicity and hippocampal neurons from Aβ_1-42_ toxicity, thereby playing a therapeutic role in these forms of human neurodegeneration (38). *Pawlosky RJ* further demonstrated that D-β-hydroxybutyrate treatment in the 3xTgAD mouse model enhances energy use in the hippocampus and reduces oxidized proteins and lipids, suggesting its neuroprotection in Alzheimer’s disease (39). Furthermore, *Yin* et al. demonstrated that two months for ketone bodies administration in a mutant amyloid precursor protein mouse model can lead to lower oxidative damage and less β-amyloid deposition, which are associated with improved learning and memory and synaptic plasticity (40). Several studies have also reported findings on the effect of KDTs on relieving human malignant gliomas (30, 41–43). For example, KDTs significantly enhance the anti-tumor effects of radiation in an intracranial bioluminescent mouse model of malignant glioma (42). In glioma stem-like cells, KDTs treatments inhibit its proliferation, increase apoptosis and attenuate its stemness by increasing reactive oxygen species production (43). These suggest that cellular metabolic alterations induced by ketone bodies may be useful as an adjuvant to the current standard of care for the treatment of human malignant gliomas. Similarly, findings on KDTs protective effects on ischemia stroke have also been wildly investigated. For example, *Min G* et al. demonstrated that classical KD can improve brain ischemic tolerance (19). And *Suzuki M* et al. demonstrated that intravenous administration of D-β-hydroxybutyrate in rats after the initiation of middle cerebral artery occlusion significantly reduces cerebral infarct area and neurological deficits (44). Clinical trials of KDTs are also on going in neurological diseases including Alzheimer’s disease, spinal cord injury, Parkinson’s disease and Rett syndrome (45–48).

#### 4.2.3 Functional effects of KDTs on metabolism

As for KDTs associated metabolic regulation, *Bough KJ* et al. suggested that KDTs dramatically affect neuronal function in the hippocampus by inducing mitochondrial biogenesis, enhancing metabolic gene expression and increasing energy reserves (49). *Shimazu T* et al. reported that D-β-hydroxybutyrate suppresses the encoding of oxidative stress resistance factors FOXO3A and MT2 through specifically inhibiting class I histone deacetylases, which is correlated with global changes in transcription (50). These analysis can provide insight into how KDTs bring neuroprotective benefits to many neurological diseases *via* regulating metabolism.

### 4.3 Research fronts in KDTs in neurological diseases

Burst keywords revealed the crucial research fronts in this field (**Figure 6**). Generally, the more recent the burst keywords, the more valuable insight into the research. Therefore, we mainly focused on the latest burst keywords and concluded them as the fronts of KDTs research. Our evaluation showed that related keywords including “Modified Atkins Diet”, “Temporal Lobe Epilepsy”, “Alzheimer’s Disease”, “Parkinson’s Disease”, “Cerebral Blood Flow”, “Neuroinflammation”, “Oxidative Stress”, “Metabolism” and “Mitochondria” were research fronts in this field and might attract wide attention of peer researchers.

#### 4.3.1 Modified Atkins Diet

Consistent with the previous discussions, our statistical data showed that other KDTs with better therapeutic performances and fewer adverse effects especially MAD have become a new research focus. They were created to avoid its side effects including body weight loss, hypoglycemia and acidosis and give full play to its strengths at the same time (11, 17).

#### 4.3.2 Application in other neurological diseases

Furthermore, our results revealed that KDTs have progressed from childhood epilepsy in the original sense to other specific neurological diseases such as temporal lobe epilepsy, adult epilepsy, Alzheimer’s disease, Parkinson’s disease, malignant glioma, amyotrophic lateral sclerosis and Huntington’s disease. Predictably, KDTs broader application might be a new investigation in the future. In addition, cerebral blood flow was extensively affected by acute cerebral hemorrhage, cerebral trauma, stroke et al. (48, 51, 52). And our results showed that the impact of KDTs on cerebral blood flow had become an emerging topic since 2017 (19, 48, 53–55). Therefore, we concluded that KDTs in such acute neurological diseases might also become an emerging research direction.

#### 4.3.3 KDTs protective mechanisms

Our results indicated that the exploration of the KDTs protective mechanism was still front. Besides the mechanisms we have discussed above at “Research Focus on KDTs in neurological diseases”, our data showed that multiple fronts such as neuroinflammation, oxidative stress, metabolic level, mitochondrial function and blood brain barrier permeability were all related to the neuroprotective role of KDTs.

Neuroinflammation is a continuous topic that occupies one of the most critical positions. Many original researches mainly focusing on neuroinflammation followed and many in-depth mechanisms were figured out (56, 57). *Youm YH* et al. reported that KDTs treatment can inhibit the NLRP3 inflammasome by preventing K (+) efflux and reducing ASC oligomerization and speck formation (57).

Oxidative stress is another essential effect of neuronal injury (47, 58). *Haces ML* et al. revealed that physiological concentrations of D-β-hydroxybutyrate are able to scavenge reactive oxygen species and hydroxyl radicals and directly reduce cellular reactive oxygen species levels, which preserve mitochondrial functioning and increase cell survival (59).

The simplest mechanism for neuroprotection is that ketone bodies serve as alternative fuels for brain metabolism, which maintain mitochondrial function, ATP production and neuronal survival. *Daniel C Shippy* et al. reported that the conversion of pyruvate to acetyl-CoA is blocked in neurodegenerative diseases. At the same time, D-β-hydroxybutyrate can provide the brain with the sole alternative source of acetyl-CoA, increasing mitochondrial acetyl-CoA, citrate and multiple tricarboxylic acids circulating metabolites (60). Since mitochondrial metabolic dysfunction is one of the crucial features to characterize neurological disease, perhaps, a change in mitochondrial status is an essential link in the metabolic mechanisms of KDTs treatment (19).

It is also worth noting that cerebral ketone body metabolism is regulated by the blood brain barrier permeability, which increases with fasting in humans. Therefore, KDTs can prevent blood brain barrier damage and promote its integrity in amyotrophic lateral sclerosis mouse model (61). *Versele R* et al. also reported that ketone bodies promotes amyloid-β_1-40_ clearance in a human *in vitro* blood brain barrier model (62). Its presence in the culture media is combined with higher monocarboxylic acid transporter-1 and glucose transporter-1 protein levels, which are essential to substances to transport through blood brain barrier (63, 64).

### 4.4 Limitations

Compared with traditional reviews, analysis based on bibliometric tools such as CiteSpace and VOSviewer provided us with better insight into the evolving research trends and relatively comprehensive data analysis. However, this study design still had certain limitations. According to our inclusion criteria, only English publications were enrolled. Therefore, some essential non-English publications might have been excluded. In addition, we only indexed the publications in the WoSCC database because of the limitations of the software CiteSpace. Although most of the researches in this field were indexed in the WoSCC database, other databases, such as PubMed and Scopus might ensure a complete representation of all available academic outputs in this field.

## 5 Conclusions

This bibliometric analysis provided an overall research profile of KDTs in neurological disease and gathered the advanced research information. The current research on KDTs mainly focused on the treatment of epilepsy, neuroprotective mechanisms and functional effects on metabolism, which were critical to improve interventions and prognosis of patients with neurological diseases. The research fronts mainly included Modified Atkins Diet, Temporal Lobe Epilepsy, Alzheimer’s Disease, Parkinson’s Disease, Cerebral Blood Flow, Neuroinflammation, Oxidative Stress, Metabolism and Mitochondria.

## Data availability statement

The original contributions presented in the study are included in the article/supplementary material. Further inquiries can be directed to the corresponding author.

## Author contributions

JY conceived the project and designed the studies. YW and JZ searched the literature together. YW and YZ analyzed the data. JY and YW drafted the figures and wrote the manuscript. All authors contributed to the article and approved the submitted version.
